# The Effect of Pets on Human Mental Health and Wellbeing during COVID-19 Lockdown in Malaysia

**DOI:** 10.3390/ani11092689

**Published:** 2021-09-14

**Authors:** Dasha Grajfoner, Guek Nee Ke, Rachel Mei Ming Wong

**Affiliations:** 1 Department of Psychology, School of Social Sciences, Heriot-Watt University, Edinburgh EH14 4AS, UK; 2 Department of Psychology, School of Social Sciences, Heriot-Watt University, Putrajaya 62200, Malaysia; g.n.ke@hw.ac.uk (G.N.K.); rach.wong29@gmail.com (R.M.M.W.)

**Keywords:** pets in Malaysia, human–animal interactions, mental health and wellbeing, lockdown, COVID-19

## Abstract

**Simple Summary:**

Pets are an integrative part of everyday life. Understanding the impact that pets have on human mental health and wellbeing, especially during periods of prolonged social isolation, is vitally important to determine whether animals can be integrated in prevention, recovery and intervention programmes to promote mental health and wellbeing. Research, with Western samples, suggests a positive impact of pets on humans; however, there is a lack of research on the effects of human–animal interactions in Southeast Asia. The aim of this study was to address this gap and to explore whether and how pets impact mental health and wellbeing in Malaysia during the COVID-19 induced movement control order (MCO). Additionally, the study explored if there was any interaction between other demographics, like age, gender, education, and pet ownership when it comes to mental health and wellbeing. The results show that in comparisons with people without animals, pet owners had significantly higher levels of mental wellbeing, in that they felt they could cope better with adverse situations and experienced significantly more positive emotions during the lockdown. On the other hand, there were no differences in levels of depression, stress, resilience, anxiety and negative emotions between the two participant groups. These results indicate that although the information about human–animal interaction is limited in Malaysia, pets can have a positive impact on some aspects of mental health and wellbeing and be actively integrated into promoting mental health and wellbeing in situations where people are socially isolated and experiencing difficulties coping with adversities or negative emotions.

**Abstract:**

The adverse impact of SARS-CoV-2 (COVID-19) on mental and physical health has been witnessed across the globe. Associated mental health and wellbeing issues include stress, social isolation, boredom, and anxiety. Research suggests human–animal interactions may improve the overall wellbeing of an individual. However, this has been less explored in Southeast Asian countries like Malaysia and the present study examined the effect of pets on the mental health and wellbeing of Malaysians during the lockdown, or movement control order (MCO), due to COVID-19 pandemic. A cross-sectional survey was carried out, with 448 Malaysian participants, who completed online assessments for psychological outcomes, psychological wellbeing, positive–negative emotions, resilience, and coping self-efficacy. Results indicate that pet owners reported significantly better coping self-efficacy, significantly more positive emotions, and better psychological wellbeing, but contrary to expectations, there was no differences on other measures. Among pet owners, cat owners reported more positive emotions and greater wellbeing than dog owners. The results show that that pets have some impact on improved psychological health of their owners and could be integrated into recovery frameworks for promoting mental health and wellbeing.

## 1. Introduction

The impact of COVID-19 and imposed restrictions on mental and physical health has been reported in a variety of studies [1,2]. More specifically high stress levels, social isolation, boredom, and anxiety have all been demonstrated both in Western [3] and Asian cultural contexts [2,4]. During the COVID-19 pandemic, the number of pets worldwide has risen exponentially and the reasons for this growth are not yet fully understood [5,6]. There are cultural and geographical differences in reference to the number and diversity of animals kept as pets; however, keeping them is a global occurrence and the research with mainly western samples shows that animals often have positive impact on human mental and physical health in general [7,8,9,10], although these findings are often challenged as inconsistent [11,12]. A number of studies investigating human–animal interactions, animal assisted activities (AAA) and animal assisted interventions (AAI) (therapy, coaching, and learning) [13] focus on the data collected in the US, UK, and European Union; the information from other parts of the world remains rather limited [14]. In Southeast Asia this research topic has been explored to an extent with samples from Hong Kong, India, China, Singapore, Thailand, South Korea [15], and Malaysia. Topics investigated included bonding between pets and pet owners in Thailand [16], AAA for undergraduate students in Singapore [17], AAI for wellbeing in older adults in India [18], and the link between attitudes towards dogs and depression [15].

Statistica [19] reports that in 2018, the number of pet dogs worldwide was 471 million, and the number of pet cats was 373 million. Within the European Union, specifically, more cats (75 million) were reported than dogs (65 million). These data suggest geographic differences in pet preferences. It is likely that Southeast Asia and particularly Malaysia, with its unique demographic structure and rich cultural and ethnic diversity, may also differ in choices regarding companion animals made in this region. According to a study in Putrajaya, which is a federal territory city in Malaysia, almost 50% of households kept pets, with 71% cats, 14% fish, and only 1.3% dogs [20]. One of the reasons for such a pet demography in this region may be in housing rules and conditions as the majority of pet owners were reported to be staying in government quarters. A higher proportion of those keeping cats in Malaysia is somehow similar to the European Union, which may also reflect a more urban lifestyle as cats are more suitable for apartment living [21]. There were more dog than cat owners taking part in our study, which does not reflect the data collected in Putrajaya; however, it still raises an interesting question: if there are any differences in mental health and wellbeing between cat and dog owners in Malaysia. A previous meta-analysis of 11 studies on this topic showed inconsistent results between the two groups of pet owners [9].

The current study investigated the impact of pets on mental health and wellbeing in Malaysia during the MCO in 2020. The situation triggered a unique opportunity to study the effect of pets on their owners during this prolonged and imposed social isolation [22,23] and the experience of loneliness [24]. The Department of Statistical Malaysia (DOSM) published the country’s demographic structure in the first quarter of 2021 showing a population in Malaysia of 32.75 million, with 16.83 million males and 15.92 million females. DOSM recorded an increase of 7.15% for the population aged 65 years and above from 2.24 million to 2.34 million between 2019 and 2020.

The novelty of the project, therefore, stems from two perspectives—limited research on the impact of social isolation due to COVID-19 on mental health and wellbeing in Malaysia and the lack of data on human–animal interactions and the effect of pets on human mental health and wellbeing in the country. Although there have been some attempts to investigate the impact of animals on human health [16,20,25,26,27,28], the studies suffer from inconsistent methodologies, small sample sizes, and are often directed towards a specific group like autistic children in special education [25] or older adults [20].

The purpose of the present study is, therefore, to explore both the structure of companion animals in Malaysia and the effect of pets on mental health and wellbeing of Malaysians during the MCO. In particular, the aims of the research project were to explore whether: (i) pet ownership significantly influences psychological wellbeing, depression, anxiety, stress, resilience, coping self-efficacy, and positive and negative emotions; (ii) types of companionship (human vs. pets) influence psychological health and wellbeing; (iii) dog and cat owners differ regards to their psychological health and wellbeing; and, finally, (iv) whether there is a relationship between pet-ownership and other demographic data (i.e., age, gender, education, marital status, and area of residence), psychological health, and wellbeing.

## 2. Materials and Methods

### 2.1. Population Study

Prior to data collection, ethical approval was obtained from the Ethics Committee at School of Sciences, Heriot-Watt University (2020-0461-1466). The single proportion formula [29] was used to estimate the optimal sample size, with a 95% confidence level and based on Malaysia’s population of 32.7 million (as estimated by the Department of Statistics Malaysia) at the time of the study. A total of 920 responses were collected, of which 224 participants were pet owners. In order to get an equal size of non-pet-owners, 224 non-pet-owners were randomly selected using SPSS’s ‘Select Cases’ function from the remaining pool of participants, making the final sample size 448 (Table 1). There were 122 dog owners and 80 cat owners participating in the study, which is inconsistent with reports from a previous study [17].

### 2.2. Procedure

The sampling frame included adults from all Malaysian states and federal territories. The survey was available in both English and Bahasa Malaysia to encourage participation. The nationwide data collection period was between June to July 2020. Due to COVID-19 health and safety measures (i.e., physical distancing requirements), data were collected remotely using the Internet and the survey application Qualtrics^®^. Posters with a QR code were placed strategically in shopping malls across various states, and third-party institutions (e.g., professional associations, NGOs, industry partners, and government agencies) assisted in questionnaire distribution by sharing the survey link with their members.

Upon accessing the URL linked to by the QR code, participants were asked to read through an information page that introduced the broad aims of the study prior to starting the survey. At the end of this page was a consent form that reminded participants of their rights, assuring anonymity and the right to withdraw from the study at any time. Upon selecting a checkbox that indicated their consent, participants were then automatically directed to the start of the survey. Aside from demographic questions, a total of five psychological measures were used to measure mental wellbeing, affect, psychological states, resilience, and coping self-efficacy. The survey was concluded with a debriefing page that provided further information about the study and links to additional resources that might be of use to the participant.

### 2.3. Materials

Outcome Measures. The Warwick-Edinburgh Mental Well-Being Scale (WEMWBS) [30] measured mental wellbeing using 14 positively phrased Likert-style scale (1 = *none of the time*, 5 = *All of the time*). The reliability of the WEMWBS (*M* = 44.32, *SD* = 10.25) in this study was 0.94, which is consistent with the original reliability (*α* = 0.91) and a similar study’s reported reliability of *α* = 0.90 [31].

The Depression, Anxiety, and Stress (DASS-21) [32] is a 21-item psychometric scale that uses a 4-point Likert-style scale (0 = *Did not apply to me at all*, 3 = *Applied to me very much, or most of the time*) to assess the symptoms of depression, anxiety, and stress. The reliability of the DASS-21 in this sample (*M_D_* = 11.97, *SD_D_* = 4.79; *M_A_* = 11.28, *SD_A_* = 4.42; *M_S_* = 12.36, *SD_S_* = 4.64) ranges from good to excellent, *α_Overall_* = 0.96, *α_D_* = 0.91, *α_A_* = 0.89, *α_S_* = 0.90. The overall reliability is consistent with the original *α* = 0.93 and *α* = 0.95 reported by a similar study [33].

The Brief Resilience Scale (BRS) [34] uses 6-items with a 5-point Likert-style scale (1 = *Strongly disagree*, 5 = *Strongly agree*; 3 items reverse scored) to measure an individual’s ability to bounce back from adversity. The BRS (*M* = 3.16, *SD* = 0.53) for this sample had a reliability of 0.52, compared to the original report’s 0.80–0.91.

The Coping Self-Efficacy (CSE) [35] measures an individual’s perceived ability to effectively cope with challenges. The CSE (*M* = 172.82, *SD* = 35.17) has 26-items in a Likert style scale (0 = *Cannot do at all*, 10 = *Certain can do*) and has a reliability of 0.52, which is lower than the original study’s reliability of α = 0.95.

The Positive and Negative Affect Schedule (PANAS) [36] used 20-items on a 5-point Likert-style scale (1 = *very slightly or not at all*, 5 = *extremely*) to measure the participant’s affective experiences and was divided into: Positive Affect (PA) and Negative Affect (NA). The scale (*M_PA_* = 30.49, *SD_PA_* = 7.47; *M_NA_* = 24.96, *SD_NA_* = 7.47) had good to excellent reliability at *α_overall_* = 0.85, *α_PA_* = 0.91, and *α_NA_* = 0.89. This is consistent with the scale’s original reliability (*α_PA_* = 0.88, and *α_NA_* = 0.87) and exceeds a similar study’s [37] reported reliability of *α_PA_* = 0.77, and *α_NA_* = 0.84.

Independent Variables. This study included several independent variables related to pet ownership, type of companionship, type of pet, and demographic characteristics. The independent variables were not measured in Likert-style scales. Pet ownership was dichotomously coded based on whether the participants owned a pet (regardless of species) (2 categories: 1 = Yes, 2 = No). Type of companionship was measured with 4 categories (1 = Pets only, 2 = Pets and humans, 3 = Humans only, 4 = None). Similarly, type of pet was measured in 4 categories (1 = Dog, 2 = Cat, 3 = Bird, 4 = Others). Participants who had other types of pets could list them in the ‘Others’ category, while participants with more than two types of pets could select the necessary options.

Age was based on a continuous measure ranging from 18–70 but later recoded into 4 categories (1 = 18–30, 2 = 31–40, 3 = 41–60, and 4 = >61 years old). Gender was coded in the conventional dichotomous measure of 1 = Male, 2 = Female. Marital status was measured based on whether participants indicated that they were 1 = Married, 2 = Single, or 3 = Others. Highest level of education attained was recoded to 2 categories (1 = Pre-University, 2 = University), with Pre-University referring to participants who have received no formal education, or any education up until college level qualifications, and University referring to participants who have received an undergraduate or up until doctorate level qualifications. Lastly, area of residence referred to the participants current living location, and measured dichotomously as 1 = Rural, 2 = Urban.

### 2.4. Performed Tests

A one-way analysis of variance (ANOVA) was conducted to evaluate any significant (*p* < 0.05) differences in the independent variables between the overall sample of pet owners and non-pet owners (Hypothesis 1). It was also conducted on data regarding the effects of type of companionship available during the MCO (with pets and humans, or with humans only) on outcome variables. Differences, if any, between dog ownership and cat ownership were assessed using multiple one-way ANOVAs, one each for each of the outcome variables of interest. To explore the last research aim, a stepwise regression was used to evaluate pet ownership and other independent variables as predictors for the outcome variables. All statistical analysis was conducted using IBM SPSS Statistics Version 27.

## 3. Results

### 3.1. Descriptives

Participants completed the survey at the end of the nation’s first Movement Control Order (MCO) and were between the ages of 18–70 (*M* = 34.75, *SD* = 11.45). Overall, females (*n* = 262) outnumbered males (*n* = 186), and majority of the sample (83.5%) were from urban places, had attained university level education (58.5%), and were married (46.7%)

### 3.2. Pet Owners vs. Non-Pet Owners

Results of the one-way ANOVA are summarized in Table 2. Descriptive statistics of pet owners and non-pet owners indicate that the mean values in pet owner group were higher in every outcome variable except negative emotions in comparison to their non-pet owner counterparts. The results indicate the presence of statistically significant differences between pet owners and non-pet owners, specifically in coping self-efficacy (f (446) = 9.42, *p* = 0.002), positive emotions (t (446) = 9.27, *p* = 0.002), and psychological wellbeing (t (446) = 4.60, *p* = 0.033).

### 3.3. Type of Companionship (Pets and Humans vs. Human Only)

Type of companionship was assessed next, specifically those with pets and humans (*n* = 208) versus human only (*n* = 186) companionship as the two groups made up most of the sample (Table 3). Mean differences indicate that those with pet and human companionship had higher scores on all outcome variables than those with human only companionship. Specifically, ANOVA revealed that differences were significant for coping self-efficacy (F (1, 392) = 9.49, *p* = 0.002) and positive emotions (F (1, 392) = 8.27, *p* = 0.004). Once again, these results indicate statistically significant differences between participants who had either pet and humans or human only companionship, although this was limited to coping self-efficacy and positive emotions only.

### 3.4. Dog vs. Cat Owners

Subsequently, dog and cat ownership were assessed, as dogs (*n* = 122) and cats (*n* = 80) were the most common pets that were owned by the participants (Table 4). Casual observation of mean scores demonstrates that cat owners, on average, scored higher than did dog owners on all outcome variables. Statistical analysis using a one-way ANOVA revealed significant differences between the two groups with regard to psychological wellbeing (F (1, 200) = 7.35, *p* = 0.007) and positive emotions (F (1, 200) = 5.22, *p* = 0.023). Of the seven outcome variables considered, these two (psychological wellbeing and positive emotions) were the only ones found to significantly differ between cat and dog owners.

### 3.5. Demographic and Pet Ownership as Predictors of the Outcome Variables

Stepwise regression results are provided in Table 5. The analysis suggests that pet ownership predicted 1% of psychological wellbeing (F (3, 444) = 8.93, *p* < 0.001), 2% of coping self-efficacy (F (1, 446) = 9.42, *p* = 0.002), and 2% of positive emotions (F (1, 446) = 9.27, *p* = 0.002). These predictors contributed less than 10% to each outcome variable but were nonetheless significant. The remaining predictors did not reach statistical significance of 0.05.

## 4. Discussion

Mental health and wellbeing have been affected during quarantine and prolonged social isolation due to COVID-19 (1). Research with Asian COVID-19 related samples report higher levels of anxiety, depression, and stress [2].

The purpose of this study was to explore whether mental health and wellbeing of pet owners during MCO in Malaysia was any different from those without pets. Results show that coping self-efficacy, psychological wellbeing, and positive emotions were significantly higher in pet owners than they were non-pet owners. Although other results were not significantly different between the two groups, there was a tendency towards pet owners scoring better on most outcome measures.

The results show that self-efficacy, which determines an individual’s perceived ability to effectively cope with challenges, was significantly higher in pet owners. This is inconsistent with two previous comparable studies, which found a trend but no significant differences in self-efficacy and self-esteem between people with and without pets in Malaysia [27] and Thailand [16]. It is crucial to keep in mind that the data in the present study were collected in an unprecedented and particularly challenging situation where study participants were experiencing a social isolation period with limited human contact and tremendous personal stress (e.g., fear of illness, dealing with present challenges to their health, employment status, and concern for their loved one’s wellbeing).

Psychological wellbeing and positive emotions were the other two outcome measures showing significant differences between the two groups. A study that explored the impact of COVID-19 on mental health and wellbeing in both pet owners and pets in the UK reported that having a companion animal may increase physical activity during social isolation, which consequently may contribute to greater wellbeing and more positive emotions [38]. The same study also argued that pets may positively affect mental health due to the social support they provide and can, therefore, moderate the effect of stressful situations that may induce anxiety and negative emotions. A similar study on the role that pets may play on human wellbeing during COVID19 in Spain reported that pets provided substantial emotional support during a 3-month period of government-mandated shelter-in-place restrictions [39].

Previous researchers have made the argument that dog owners may obtain greater benefits from their canine companions, due to the physical activity and increased social contact resulting when dog walking [40,41,42,43]. Although there were more dog owners that participated in the present study, the results showed that cat owners showed greater psychological wellbeing and reported more positive emotions during the MCO than did dog owners. Perhaps interactions with cats, petting and the feedback they provide, moderates emotional regulation more that physical activity linked to owning a dog.

Out of 224 pet owners, a majority of them (208) shared their household with other humans during the MCO. Although there is some information on self-isolation and social distancing [1], none of the studies actually looked at pets with humans vs. human company only [1,5]. With that in mind this study explored whether types of companionship, human vs. pets and human, influence psychological health and wellbeing. The result show that all measures, except negative emotions, are higher in pet and human companionship groups, with coping self-efficacy and positive emotions being significantly different. Previous research on animal assisted visitation programmes also showed that participants who interacted with just a human handler, as opposed to therapy dog or dog and handler, produced lower scores on a variety of outcome measures, including wellbeing, anxiety and stress [11].

Looking at the interaction effect of demographic data and pet ownership as predictors of psychological wellbeing, depression, anxiety, stress, resilience, coping self-efficacy, positive emotions, and negative emotions, it showed that pet ownership predicts psychological wellbeing together with age and the area of residence. Although previous research in Malaysia [27] did not consider demographic data, over 80% of pet owners, who showed a tendency for higher self-esteem and self-efficacy, had a diploma or university level education.

Positive emotions are also predicted by pet ownership and education, with those pet owners who are older and have a higher education also achieving higher scores on Positive Affect part of PANAS. Education, in particular, seemed to be an important confounding variable, as pet owners who are more educated were doing better, which is in line with some previous research [11].

Methodological challenges in human–animal interaction research touch on sampling, study design, randomization, and the problem of heterogeneity of both animals and humans involved [8,14]. While there was no control over the type of pets participants shared their life with at the time of completing the tests, this study aimed to match participants without pets to pet owners by using inclusion criteria of age, education, gender, and area of living.

Very few participants of age 65 and above participated in the study and so had to be removed from the final analysis. This is one of the limitations of the study, as older adults can be particularly vulnerable in situations that cause prolonged social isolation due to other health issues and may benefit the most from companion animals [27,44,45]. Therefore, additional research, focusing on the benefits on mental health and wellbeing in older adults that are experiencing social isolation regardless of COVID-19 restrictions would enhance the results of this study and further develop our understanding of the value sharing our lives with companion animals.

## 5. Conclusions

Our research aims and hypotheses have explored different aspects of the impact pets have on human mental health and wellbeing in Malaysia. The novelty of the study was primarily on exploring the topic in a country like Malaysia, with an increasing number of pets and limited research. Previous research on the effects of pets on mental health and wellbeing varies and the results of our study are in line with previous findings. Some variables seem to be associated with pet ownership, whilst others, contrary to our expectations, do not show significant relationship.

The results of the study support the argument that pets can have a positive impact on some aspects of mental health and wellbeing in very challenging times, where contact with other humans may not be possible. Consequently, companion animals and other forms of animal assisted activities and interventions can be considered in the context of any mental health and wellbeing recovery programs and explored in other situations and with other groups of participants, who could in different circumstances experience lack of human contact and social isolation.

## Figures and Tables

**Table 1:** Participant characteristics by pet ownership.

		Pet Owners		Non-Pet Owners	
Demographic	Category	*n*	%	*n*	%
Age	18–30	102	45.5%	96	42.9%
	31–40	58	25.9%	71	31.7%
	41–60	57	25.4%	52	23.2%
	>61	7	3.1%	5	2.2%
Gender	Male	88	39.3%	98	43.8%
	Female	136	60.7%	126	56.3%
Marital Status	Married	99	44.2%	110	49.1%
	Single	122	54.5%	112	50.0%
	Other	3	1.3%	2	0.9%
Education ^1^	Pre-university	103	46.0%	83	37.1%
	University	121	54.0%	141	62.9%
Area	Rural	39	17.4%	35	15.6%
	Urban	185	82.6%	189	84.4%

^1^ Under Education, Pre-university refers to participants who have either no formal education or have attained a college level qualification. University refers to participants who have attained an undergraduate to doctorate level qualification.

**Table 2:** Results of ANOVA based on pet ownership.

	Outcome Variables	Pet Owners		Non-Pet Owners		f
		M	SD	M	SD	
1	Psychological Wellbeing	45.35	10.58	43.28	9.81	4.60 *
2	Depression	24.36	9.66	23.54	9.50	0.82
3	Anxiety	22.56	8.79	22.55	8.91	0.00
4	Stress	24.92	9.34	24.51	9.23	0.22
5	Resilience	19.09	3.19	18.87	3.17	0.53
6	Coping self-efficacy	88.93	16.00	83.88	18.74	9.42 **
7	Positive emotions	31.56	7.63	29.43	7.16	9.27 **
8	Negative emotions	24.85	7.95	25.08	6.98	0.10

* *p* < 0.05, ** *p* < 0.005.

**Table 3:** Results of one-way ANOVA based on pet and human companionship versus human only companionship.

Outcome Variables		Pets and Humans (*n* = 208)		Humans Only (*n* = 186)		SS	df	MS	F
		M	SD	M	SD				
1	Psychological Wellbeing	45.31	10.71	43.58	9.74	292.88	1, 392	292.88	2.78
2	Depression	24.72	9.77	22.91	9.22	320.69	1, 392	320.69	3.54
3	Anxiety	22.66	8.81	22.13	9.05	28.05	1, 392	28.05	0.35
4	Stress	25.07	9.30	24.05	9.47	100.87	1, 392	100.87	1.15
5	Resilience	19.11	3.28	18.99	3.16	1.31	1, 392	1.31	0.13
6	Coping self-efficacy	88.88	15.99	83.50	18.68	2842.57	1, 392	2842.57	9.49 **
7	Positive emotions	31.50	7.73	29.37	6.87	445.09	1, 392	445.09	8.27 **
8	Negative emotions	24.73	8.07	24.78	6.86	0.23	1, 392	0.23	0.00

**Note:** There was insufficient data for pet’s only companionship. ** *p* < 0.005.

**Table 4:** Results of one-way ANOVA based on dog and cat ownership.

Outcome Variables		Dog Owners (*n* = 122)		Cat Owners (*n* = 80)		SS	df	MS	F
		M	SD	M	SD				
1	Psychological Wellbeing	43.80	10.24	47.94	11.13	825.82	1, 200	825.82	7.35 *
2	Depression	23.56	8.37	25.08	10.84	111.28	1, 200	111.28	1.25
3	Anxiety	21.90	7.92	23.05	9.32	63.72	1, 200	63.72	0.88
4	Stress	23.77	8.28	25.93	10.40	224.28	1, 200	224.28	2.66
5	Resilience	19.11	2.73	19.28	3.67	1.37	1, 200	1.37	0.14
6	Coping self-efficacy	88.29	14.33	89.29	19.47	48.36	1, 200	48.36	0.18
7	Positive emotions	30.70	6.60	33.15	8.63	290.80	1, 200	290.80	5.22 *
8	Negative emotions	24.86	7.26	25.15	8.94	4.05	1, 200	4.05	0.06

* *p* < 0.05.

**Table 5:** Results of stepwise regression (only significant predictors are shown).

Outcome		Predictor	r^2^	B	SE	Beta	T
1	Psychological wellbeing	Age	0.03	1.69	0.50	0.16	3.40 ***
		Area of residence	0.02	3.42	1.28	0.12	2.68 *
		Pet ownership	0.01	−2.09	0.95	−0.10	−2.21*
2	Depression	Age	0.05	−2.20	0.46	−0.22	−4.75 ***
3	Anxiety	Age	0.04	−1.79	0.43	−0.19	−4.17 ***
4	Stress	Age	0.03	−1.59	0.45	−0.16	−3.50 ***
5	Resilience	Age	0.06	0.57	0.18	0.17	3.24 **
		Gender	0.01	−0.77	0.29	−0.12	−2.62 *
		Marital Status	0.01	−0.77	0.32	−0.13	−2.38 *
6	Coping self-efficacy	Pet ownership	0.02	−5.34	1.62	−0.15	−3.30 **
		Area of residence	0.02	4.98	2.23	0.11	2.23 *
		Education	0.01	4.42	1.70	0.12	2.60 *
		Age	0.01	2.15	0.86	0.12	2.50 *
		Gender	0.01	3.40	1.65	0.10	2.07 *
7	Positive emotions	Pet ownership	0.02	−2.29	0.69	−0.15	−3.29 **
		Education	0.01	1.99	0.71	0.13	2.79 **
		Age	0.01	0.89	0.37	0.11	2.44 *
8	Negative emotions	Age	0.04	−1.13	0.42	−0.15	−2.69 *
		Marital Status	0.01	1.59	0.78	0.11	2.05 **

* *p* < 0.05, ** *p* < 0.005, *** *p* < 0.001.

## Data Availability

All relevant data is contained within the article.
